# BfvR, an AraC-Family Regulator, Controls Biofilm Formation and pH6 Antigen Production in Opposite Ways in *Yersinia pestis* Biovar Microtus

**DOI:** 10.3389/fcimb.2018.00347

**Published:** 2018-10-02

**Authors:** Haihong Fang, Lei Liu, Yiquan Zhang, Huiying Yang, Yanfeng Yan, Xiaojuan Ding, Yanping Han, Dongsheng Zhou, Ruifu Yang

**Affiliations:** ^1^State Key Laboratory of Pathogen and Biosecurity, Beijing Institute of Microbiology and Epidemiology, Beijing, China; ^2^Division of Biology, Beijing Academy, Beijing, China; ^3^Department of Blood Transfusion, Wuhan General Hospital of PLA, Wuhan, China; ^4^School of Medicine, Jiangsu University, Zhenjiang, China; ^5^Department of Microbiology, Anhui Medical University, Hefei, China

**Keywords:** *Yersinia pestis*, BfvR, biofilm, virulence, transcriptional regulation

## Abstract

Biofilm formation is critical for blocking flea foregut and hence for transmission of *Y. pestis* by flea biting. In this study, we identified the regulatory role of the AraC-family transcriptional regulator BfvR (YPO1737 in strain CO92) in biofilm formation and virulence of *Yersinia pestis* biovar Microtus. Crystal violet staining, *Caenorhabditis elegans* biofilm assay, colony morphology assay, intracellular c-di-GMP concentration determination, and BALB/c mice challenge were employed to reveal that BfvR enhanced *Y. pestis* biofilm formation while repressed its virulence in mice. Further molecular biological assays demonstrated that BfvR directly stimulated the expression of *hmsHFRS, waaAE-coaD*, and *hmsCDE*, which, in turn, affected the production of exopolysaccharide, LPS, and c-di-GMP, respectively. In addition, BfvR directly and indirectly repressed *psaABC* and *psaEF* transcription, respectively. We concluded that the modulation of biofilm- and virulence-related genes by BfvR led to increased biofilm formation and reduced virulence of *Y. pestis* biovar Microtus.

## Introduction

*Yersinia pestis*, which causes severe and even fatal zoonotic diseases, has been responsible for the three plague pandemics throughout human history. *Y. pestis* can form biofilm not only *in vitro*, but also in the infected fleas that leads to a blockage in the proventriculus and enhances the flea-borne transmission (Hinnebusch et al., 1996). Studies on biofilm formation in *Y. pestis* have focused on the synthesis and degradation of: (i) extracellular matrix (Bobrov et al., 2008); (ii) intracellular secondary messengers, such as 3′,5′-cyclic diguanylic acid (c-di-GMP) (Kirillina et al., 2004; Bobrov et al., 2011); and (iii) lipopolysaccharide (LPS) (Knirel and Anisimov, 2012). Transcription regulation can also modulate the expression of biofilm-related genes through multiple cellular pathways (Sun et al., 2009; Sun Y. C. et al., 2012; Willias et al., 2014, 2015).

Bacterial exopolysaccharide (EPS) is the primary component of the biofilm extracellular matrix. The *hmsHFRS* operon, located in the 102-kb *pgm* locus, coordinates the synthesis and transport of EPS in *Y. pestis* (Bobrov et al., 2008). The secondary signaling molecule c-di-GMP promotes EPS production in *Y. pestis*. This signaling molecule is catalyzed by diguanylate cyclases (DGC) and degraded by phosphodiesterase (PDE). In *Y. pestis*, DGC is encoded by two genes, *hmsT* and *hmsD* (derived from the three-gene operon *hmsCDE*); while PDE is encoded by *hmsP* (Kirillina et al., 2004). In general, a high concentration of intracellular c-di-GMP can stimulate biofilm formation and interfere with virulence (Hengge, 2009). DGC and PDE inversely regulate biofilm formation, which is dependent on control of HmsHFRS-based poly-β-1, 6-N-acetylglucos-amine synthesis in *Y. pestis* (Bobrov et al., 2011).

LPS also plays a vital role in biofilm formation and is synthesized by a three-gene operon *waaAE*-*coaD*, containing genes *waaA, waaE*, and *coaD*, in *Y. pestis*. Deletion of *waaA*, which encodes a 3-deoxy-D-manno-octulosonic acid transferase involved in the synthesis of LPS (Tan and Darby, 2005), results in a biofilm-defective phenotype in *Y. pestis* (Liu et al., 2014).

It was reported that the expression of 214 genes significantly increased in the flea compared with all *in vitro* growth conditions, including *y2570* (YPO1737 in CO92 and *bfvR* in Microtus strain 91001). Whole-genome microarrays in *Y. pestis* KIM6+ showed that *y2570* had significantly higher expression levels in the flea gut but not the rat bubo (Vadyvaloo et al., 2010). We predicted and identified that *bfvR*, located between the bases 1,639,340 and 1,639,726 on the genome of Microtus strain 91001, can participate in biofilm formation *in vivo* (Song et al., 2004; Mao et al., 2016). In this research, we also studied the regulation mechanisms on biofilm formation for the mentioned genes by BfvR in *Y. pestis*.

Moreover, we found *bfvR* could inhibit the virulence of BALB/c mice in 91001. The AraC family transcription regulator typically binds to the target DNA and regulates bacterial virulence by sensing small molecule inducers such as urea, bicarbonate, or cellobiose etc. The molecular inducers were abundant at the sites where the bacterial pathogen colonizes and damages its host (Yang et al., 2011).

The protein Psa, known as pH 6 antigen, is one of the surface proteins involved in adhesion to the host cellular surface during initiation of *Y. pestis* pathogenesis. Psa proteins result from the polymerization of single PsaA pilin subunits (Bao et al., 2012), and the assembly of pH 6 antigen is mediated by the secretion of PsaB and PsaC, which constitutes the chaperone/usher machinery on the cell surface (Price et al., 1995). PsaABC is highly expressed following a temperature shift from 26 to 37°C and acidic media, while PsaEF is responsible for the transcriptional activation of *psaABC* (Yang and Isberg, 1997). pH 6 antigen mediates the entry of *Y. pestis* into human pulmonary epithelial cells (Liu et al., 2006; Galvan et al., 2007) and promotes the delivery of Yops (effectors of the plasmid pCD1-encoded type III secretion system) into target host cells via cell-to-cell contact between *Y. pestis* and eukaryotic cells (Felek et al., 2010). pH 6 antigen does not strengthen the attachment to mouse macrophages, however, the resistance to phagocytosis can be developed independent of *Yersinia* outer proteins and capsule antigen in *Y. pestis* KIM5 (Huang and Lindler, 2004). However, loss of Psa has no effect on the virulence of *Y. pestis* strain 231 following subcutaneous challenge of naive and pH 6 antigen-immunized mice (Anisimov et al., 2009). In our opinion, the role of pH 6 antigen in *Y. pestis* virulence appears to be dependent on the strains tested and the animals challenged. In our study, we showed that by decreasing the transcription of *psaABC* and *psaEF*, BfvR could influence the survival of mice infected with strain 91001.

There are 7–19 AraC- or AraC-like-family transcription regulatory genes deposited in the GenBank database from *Yersinia* genomes. *Y. pestis* biovar Microtus strain 91001, Orientalis strain CO92, and Medievalis strain KIM D27 possess 19, 13, and 14 AraC family transcription regulators, respectively. YbtA, a previously reported AraC family transcription regulator in *Y. pestis*, can promote the transcription of *psn* (receptor for yersiniabactin) and *irp2* (yersiniabactin biosynthesis gene), but inhibit the transcription of *ybtA* itself (Fetherston et al., 1996). MarA can affect bacterial drug sensitivity (Gillette et al., 2000), both MarA47 and MarA48 in the KIM strain of *Y. pestis* can regulate drug sensitivity and virulence (Lister et al., 2010). LcrF, similar to ExcA (an AraC family transcription regulator in *Escherichia coli*), can also regulate the transcription of the *Ysc*-*Yop* genes of type III secretion system in *Y. pestis* (King et al., 2013; Li et al., 2014). However, none of the AraC family transcription regulators have been reported to regulate biofilm formation in *Y. pestis*.

In this study, we firstly revealed the regulation of biofilm formation by BfvR in *Y. pestis*. In addition, our findings offer insights into the coordination of virulence and biofilm formation in *Y. pestis*.

## Materials and methods

### Bacterial strains and mutant preparation

The wild-type (WT) *Y. pestis* strain 91001 is avirulent in humans but highly lethal to mice (Zhou et al., 2004). The entire coding region of *bfvR* was replaced with the kanamycin resistance cassette using the one-step inactivation method based on the lambda Red recombination system (Datsenko and Wanner, 2000) and the *Y. pestis bfvR* null mutant was designated as Δ*bfvR*. All primers used in this study were listed in Table S1.

A PCR-generated DNA fragment containing the *bfvR* coding region with its 609-bp upstream promoter-proximal region and 267-bp downstream transcriptional terminator region was cloned into the pACYC184 vector (GenBank accession number X06403). The recombinant plasmid was introduced into Δ*bfvR*, yielding the complementary strain *C-bfvR*.

For *psaABC*-related gene regulation experiments, supplemented BHI (sBHI) broth [3.7% Bacto brain heart infusion (BD Biosciences), 0.5% Oxoid yeast extract, 2.5 mM CaCl_2_, 0.2% xylose, pH 6.0] was used for bacterial cultivation (Lindler et al., 1990). An overnight bacterial culture with an optical density (OD620) value of about 1.0 was diluted 1:20 into fresh sBHI for further growth at 26°C with 230-rpm shaking. The cell culture with an OD620 of about 0.6 was transferred to 37°C for a further 3-h culture prior to the harvesting of cells.

For *bfvR* gene regulation and phenotypic experiments, an overnight cell culture in Luria–Bertani (LB) broth with an OD620 of about 1.5 was immediately diluted 1:20 into fresh LB broth for the second generation cultivation at 26°C to reach an OD620 of about 1.0 and the culture was stored at 4°C for up to 24 h. The culture was also diluted 1:20 into fresh LB broth for the third cultivation at 26°C for 6–8 h to reach an OD620 of about 1.0.

### Biofilm-related assays

Four different methods (Fang et al., 2013, 2015) of biofilm-related assays were employed: (i) Crystal violet staining of biofilms, the *in vitro* biofilm masses attached to the well walls were stained by crystal violet when bacteria were grown in polystyrene microtiter plates; (ii) *Caenorhabditis elegans* biofilm assay, the nematode eggs were inoculated onto *Y. pestis* lawns and the percentages of fourth-stage larvae and adults (L4/adult) of *C. elegans* were determined for evaluating the bacterial ability to produce biofilms; (iii) the colony morphology assay, the rugose colony morphology of bacteria grown on LB agar plates was observed at different times for assessing the bacterial ability to synthesize biofilm matrix exopolysaccharide; and (iv) determination of intracellular c-di-GMP levels by a chromatography-coupled tandem mass spectrometry method (Spangler et al., 2010).

### Murine infection model

All animal experiments were carried out in accordance with the principles of the Basel Declaration and recommendations of the Guidelines for Welfare and Ethics of Laboratory Animals of China, the Committee on Animal Research of the Academy of Military Medical Sciences. The protocol was approved by the Committee on Animal Research of the Academy of Military Medical Sciences. Bacterial cultures were washed twice with PBS (pH 7.2), and then subjected to serial 10-fold dilutions with PBS. Appropriate dilutions were plated onto He's agar plates (1 L powder containing pig blood peptone 12 g, K_2_HPO_4_1 g, NaCl 4 g, and agar 13 g, LandBridge,China) to calculate the number of colony-forming units (CFU). For each strain tested, 0.1 ml of the 10^3^ CFU/ml bacterial suspensions were inoculated by subcutaneous injection at the inguinal region or by intravenous injection via the tail vein into each of 10 female BALB/c mice (aged 6 to 8-week-old). The numbers of mice that died at specified times were recorded and used to create a survival curve with the GraphPad Prism 5.0 software. *P*-values were calculated with the log-rank (Mantel–Cox) test and the Gehan–Breslow–Wilcoxon test affiliated in this software. *P* < 0.01 was considered to indicate statistical significance.

### Primer extension (PE) assay

For the PE assay (Ghosh et al., 1978; Sun F. et al., 2012; Zhang et al., 2013), an oligonucleotide primer complementary to a portion of the RNA transcript of each indicated gene was used to synthesize cDNAs. About 10–30 μg of total RNA from each strain was annealed with 1 pmol of [γ−^32^P] end-labeled reverse primer using a Primer Extension System (Promega) in accordance with the manufacturer's instructions. The same labeled primer was also used for sequencing with the fmol® DNA Cycle Sequencing System (Promega). The PE products and sequencing DNAs were concentrated and analyzed in a 6% polyacrylamide/8 M urea gel. The results were detected by autoradiography (Kodak film) and then analyzed.

### Quantitative RT-PCR

Gene-specific primers were designed to produce amplicons for the target genes. Contaminating DNA in the RNA samples was removed using the Ambion DNA-free™ Kit (Applied Biosystems). cDNAs were generated using 5 μg of RNA and 3 μg of random hexamer primers. Real-time PCR was performed using the LightCycler system (Roche) and the SYBR Green master mix (Takara) (Zhan et al., 2008; Sun et al., 2014). Based on the standard curves of 16S rRNA gene expression, the relative mRNA level was determined by calculating the threshold cycle (Ct) of target genes via the classic 2-ΔΔCT method. Negative controls used cDNA generated without reverse transcriptase as templates. Reactions containing primer pairs without template were also included as blank controls. The *16S rRNA* gene was used as an internal control for normalization.

### LacZ reporter fusion and β-galactosidase assay

The promoter-proximal DNA region of each gene tested was prepared by PCR with Takara ExTaq DNA polymerase using *Y. pestis* strain 91001 genomic DNA as the template. This purified fragment was then cloned directionally into the *Hin*dIII–*Bam*HI site of the transcriptional fusion vector pRW50 (El-Robh and Busby, 2002) that contained a promoterless *lacZ* reporter gene. The clone was verified by DNA sequencing. Each *Y. pestis* strain tested was transformed with the recombinant plasmid and the empty plasmid pRW50 as a negative control. β-galactosidase activity in extracts was measured from the cells cultivated as described above according to the β-Galactosidase Enzyme Assay System (Promega) (Sun F. et al., 2012; Zhang et al., 2013).

### Preparation of 6×his-tagged BfvR protein

The preparation of purified BfvR protein was performed as previously described (Heroven and Dersch, 2006). The entire coding region of *bfvR* was amplified from *Y. pestis* strain 91001 and cloned directionally into the *Bam*HI and *Hin*dIII sites of plasmid pColdI (Qing et al., 2004) (Novagen, GenBank accession number AB186388). The recombinant plasmids were transformed into *E. coli* BL21 (DE3) cells (Novagen). Expression of His-BfvR protein was induced by the addition of 1 mM or 2 mM isopropyl-beta-D-thiogalactoside at 16°C overnight. His-BfvR was purified under native conditions using QIAexpressionist™ Ni-NTA affinity chromatography (Qiagen). The purified eluted protein was concentrated with an Amicon Ultra-15 column (Millipore) to a final concentration of 0.5–0.7 mg/ml in storage buffer containing 10 mM Tris-HCl, 1 mM EDTA, 0.1 mM DTT, and 5 mM β-mercaptoethanol (pH 7.0) plus 20% glycerol. The protein purity was verified by sodium dodecyl sulfate–polyacrylamide gel electrophoresis with Coomassie brilliant blue staining.

### Electrophoretic mobility shift assay (EMSA)

For EMSA, we have made minor modifications according to the Hellman's method (Hellman and Fried, 2007). Promoter-proximal DNA region was prepared by PCR amplification. The 5′ end of the DNA was labeled using [γ-^32^P] ATP and T4 polynucleotide kinase. DNA binding was performed in a 10 μl volume containing binding buffer [100 μM MnCl_2_, 1 mM MgCl_2_, 0.5 mM DTT, 50 mM KCl, 10 mM Tris-HCl (pH 7.5), 0.05 mg/ml sheared salmon sperm DNA, 0.05 mg/ml BSA, and 4% glycerol], labeled DNA (1,000 to 2,000 c.p.m/μl), and increasing amounts of His-RovA or His-BfvR. Two control reactions were included: one contained the specific DNA competitor (unlabeled promoter DNA regions; cold probe), while the other was the non-specific protein competitor (rabbit anti-F1-protein polyclonal IgG antibody). After incubation at room temperature for 30 min, the products were loaded onto a native 4% (w/v) polyacrylamide gel and electrophoresed in 0.5 × Tris-borate buffer containing 100 μM MnCl_2_ for 30 min with 220 V at 8°C. Radioactive species were detected by autoradiography.

### Experimental replicates and statistical methods

For real-time RT-PCR, the β-galactosidase activity assay, crystal violet staining of biofilms, the determination of L4/adult nematodes, c-di-GMP experiments and murine infection were performed with at least three independent bacterial cultures/lawns, and values were expressed as the mean ± standard deviation. The paired Student's *t*-test was performed to determine significant differences; *P* < 0.01 was considered to indicate statistical significance. For primer extension, EMSA, and colony morphology observations, representative data from at least two independent biological replicates were shown.

## Results

### Involvement of BfvR in biofilm formation and virulence in mice

To investigate the potential role of BfvR in biofilm formation, the phenotypes of relevant mutant strains were analyzed. We selected four experimental strains: (i) wild-type (WT); (ii) *bfvR* mutant strain (Δ*bfvR*); (iii) pACYC184-Δ*bfvR* complementary strain (*C-bfvR*); and (iv) a strain containing a mutation in the biofilm-related gene *hmsS* (Δ*hmsS*), a typical biofilm negative strain.

**Figure 1:**
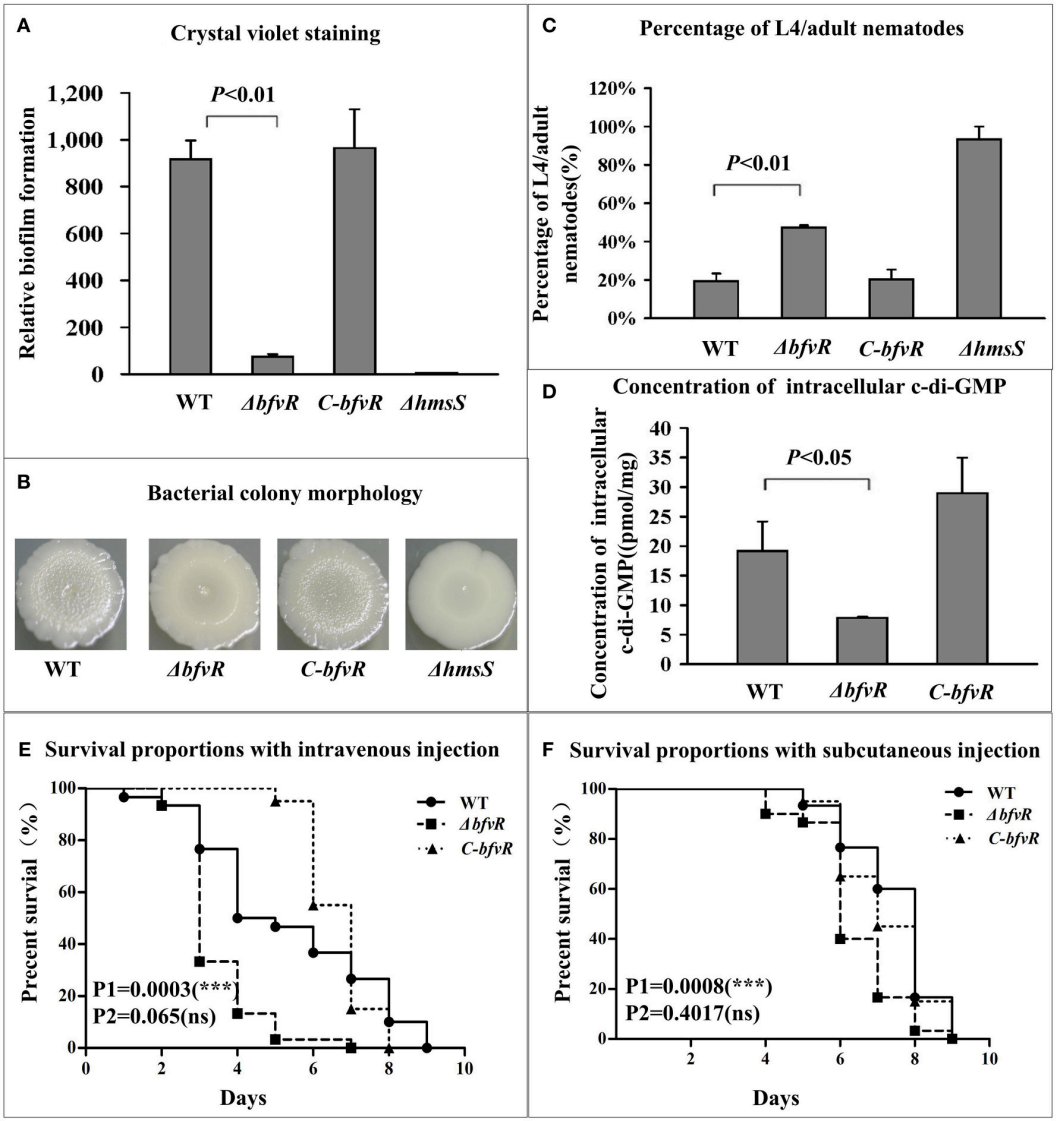
BfvR-mediated phenotypes. **(A)** Crystal violet staining of biofilms. Cultured for 48 h in 24-well polystyrene dishes and evaluated by the OD570/OD620 value. **(B)** Bacterial colony morphology. Spotted on the LB agar with the glycerol stocks and cultured for 1 week at 26°C and then analyzed. **(C)** Biofilms on nematodes. Calculated by the percentage of L4/adults after 48-h feeding with different strains. **(D)** Intracellular c-di-GMP concentrations. Isolated c-di-GMP from the logarithmic bacteria and determined the concentrations by UPLC-MS/MS. **(E,F)** Virulence in BALB/c mice. Infected with 50-150 CFU by intravenous injection via the tail vein **(E)** and the subcutaneous injection at the inguinal region **(F)** and evaluated the virulence by the survival curve and P value with the GraphPad Prism 5.0 software. This is a representative experiment. P1 and P2 represented the *P* values between WT and Δ*bfvR* or *C-bfvR*, respectively. *** Shows significant difference, ns indicates no significant difference.

Deletion of *bfvR* had no effect on the growth in LB (Figure S1) but led to a dramatic reduction in biofilm formation in *Y. pestis* strain 91001. Compared with the WT and *C-bfvR* strains, the Δ*bfvR* mutant strain showed less crystal violet staining (Figure 1A), with smoother colony morphology (Figure 1B), similar to the Δ*hmsS* mutant. After the nematode eggs were fed with Δ*hmsS* strain, 93% of the eggs developed into L4/adult nematodes, compared with 19.3, 47.3, and 20.3% for the WT, Δ*bfvR*, and *C-bfvR* strains, respectively (Figure 1C), indicating *bfvR* might affect *C. elegans* development. In general, when fed with WT strain, about 20% or fewer larvae grew and developed into L4/adult nematodes (Zhou and Yang, 2011; Fang et al., 2013); however, the exact ratio of nematode adults did not directly correlate with the biofilm formation ability of *Y. pestis*. The concentration of intracellular c-di-GMP was quantitatively detected by UPLC-MS/MS analysis (Spangler et al., 2010). Lower production of cellular c-di-GMP was observed in strain Δ*bfvR* (11.45 nmol/μg) compared with the WT (19.68 nmol/μg) and *C-bfvR* (32.70 nmol/μg) strains (Figure 1D). These phenotypes suggested that deletion of *bfvR* resulted in an obvious reduction in biofilm/c-di-GMP production.

Wild type bacteria, the BfvR mutant and the complemented strain were used to infect mice through both subcutaneous injection and intravenous injection. This was repeated three times independently for WT and the BfvR mutant, and repeated two times for the complemented strain. Finally, we calculated all the results and drew the survival curves with Graphpad 5.0. Compared with WT, Δ*bfvR* displayed a significant increase in virulence in BALB/c mice after intravenous (*P* = 0.0003) or subcutaneous (*P* = 0.0008) injection. Moreover, compared to WT, the complementary effect of *C-bfvR* also showed no significant differences during the intravenous (*P* = 0.065) or subcutaneous (*P* = 0.4017) routes of infection (Figures 1E,F).

Taken together, BfvR could enhance biofilm formation and inhibit the virulence of *Y. pestis* strain 91001 in BALB/c mice.

### BfvR acts as an activator of EPS and c-di-GMP

In order to reveal the mechanisms by which BfvR modulated the synthesis of EPS, we analyzed expressions of the related genes including *hmsH* and *waaA*. The genes encoding DGC and PDE, namely *hmsC, hmsT*, and *hmsP* in *Y. pestis*, were also evaluated by qRT-PCR, PE, β-galactosidase activity assays and EMSA.

**Figure 2:**
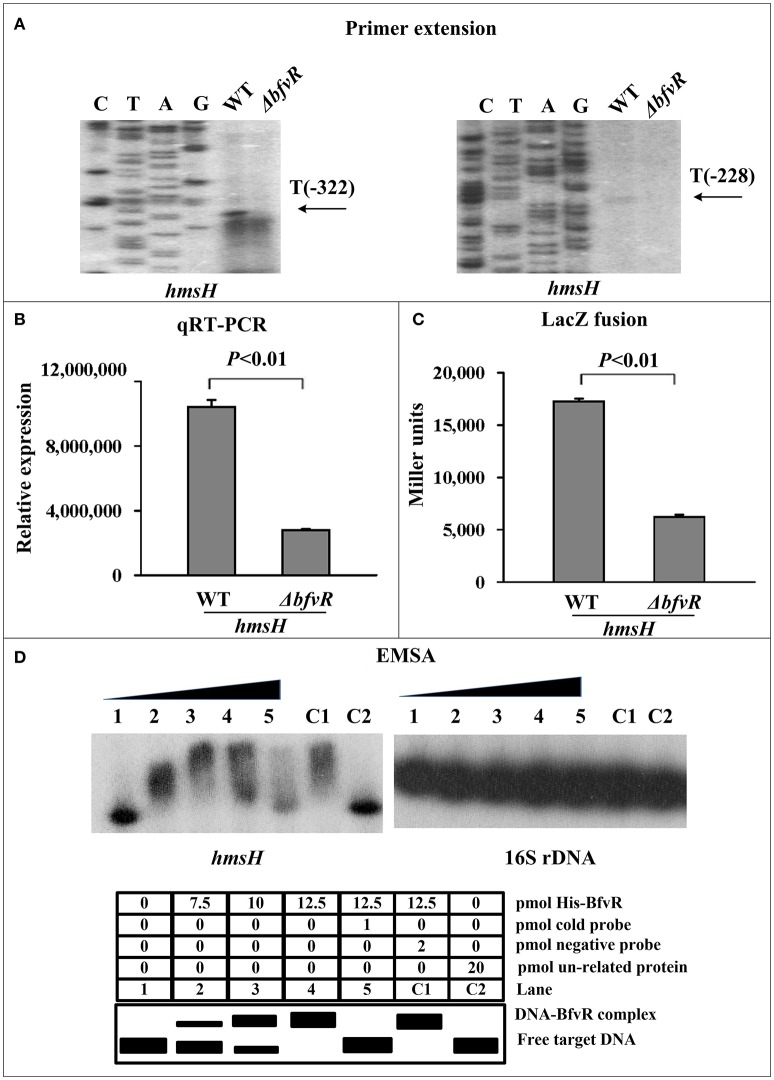
Regulation of *hmsH* transcription by BfvR. Lanes G, A, T, and C represented Sanger sequencing reactions. Symbols + and – indicated the nucleotide positions downstream and upstream of the translation start site of target gene, respectively. **(A)** Primer extension. A(−128) represents the transcription start site of *hmsH*. **(B)** Quantitative RT-PCR. The relative mRNA expression was determined by calculating the threshold cycle (Ct) of *hmsH* via the classic 2-ΔΔCt method through standardized by the 16S rRNA gene. **(C)** LacZ fusion. LacZ reporter fusion with the promoter-proximal region (−451…+103) of *hmsH* and β-galactosidase in cellular extracts were determined. **(D)** EMSA. Purified His-BfvR protein bound to the promoter-proximal fragment of *hmsH* labeled radioactively and the retarded DNA band were shown in the amount-dependent manner.

BfvR directly promoted *hmsH* expression. The mRNA levels of *hmsH* in WT and Δ*bfvR* strains were analyzed by primer extension. The levels of mRNA from the two transcriptional start sites (Sun F. et al., 2012) were found to be lower in Δ*bfvR* than in the WT strain (Figure 2A). Quantitative real-time PCR (qRT-PCR) assay indicated a significant reduction in relative mRNA expression in Δ*bfvR* compared with the WT strain (Figure 2B). A β-galactosidase activity assay also indicated that the promoter activity of *hmsH* (Figure 2C) was attenuated in Δ*bfvR* relative to the WT. EMSA showed that His-BfvR could bind to the promoter-proximal regions of *hmsH* in a dose-dependent manner (Figure 2D). These results indicated that BfvR in *Y. pestis* strain 91001 could promote biofilm formation by directly stimulating *hmsH* expression.

**Figure 3:**
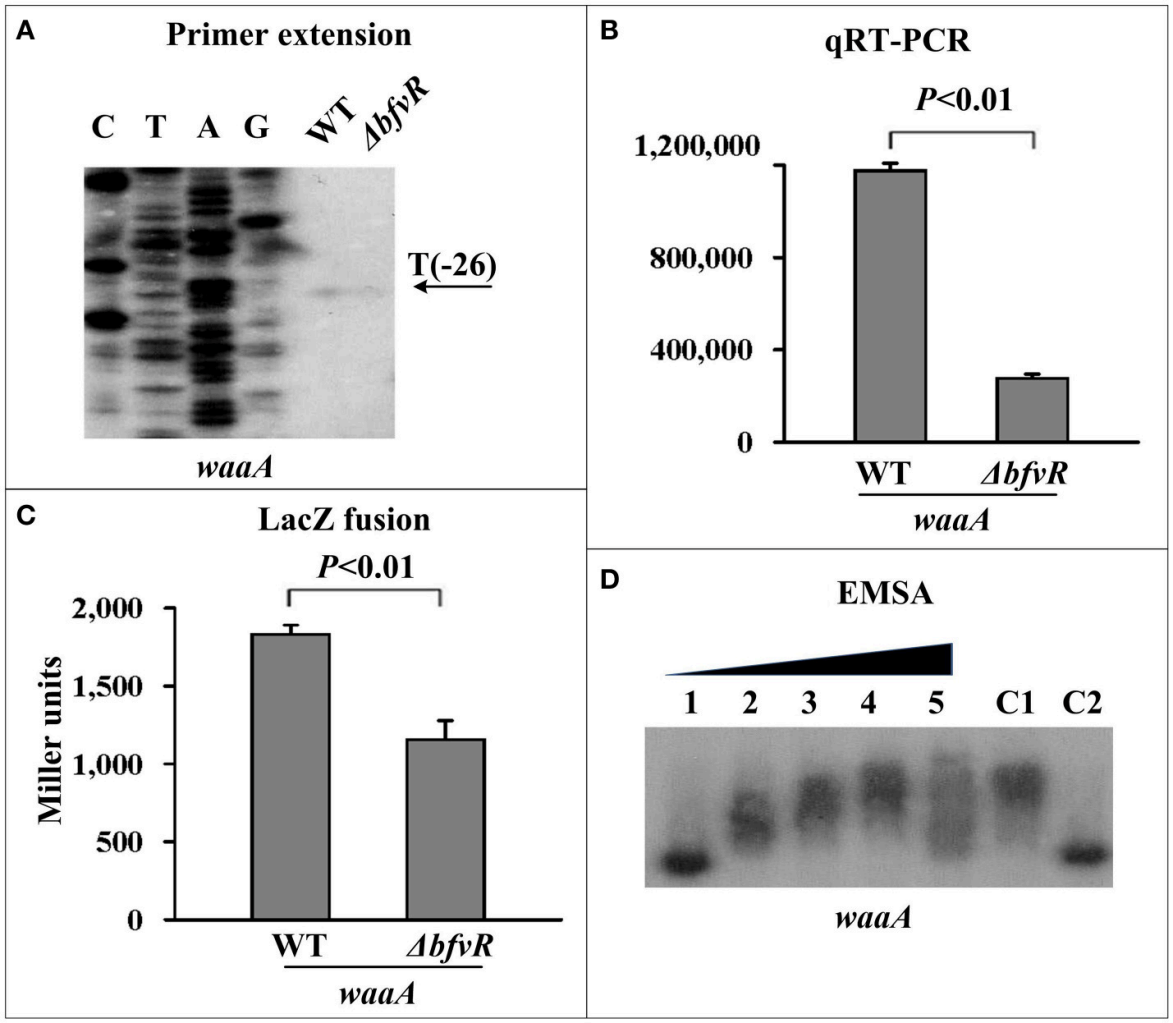
Regulation of *waaAE-coaD* by BfvR. Reference to Figure 2 for the explanation of primer extension **(A)**, quantitative RT-PCR **(B)**, LacZ fusion **(C)**, and EMSA **(D)**. The promoter-proximal region from −574 to +62 was amplified.

BfvR directly induced *waaAE-coaD* expression. The transcription of *waaA* (Liu et al., 2014), the first gene in the *waaAE-coaD* operon, was investigated in both WT and Δ*bfvR* strains. Both primer extension (Figure 3A) and qRT-PCR assays (Figure 3B) revealed a significant reduction in *waaAE-coaD* expression in Δ*bfvR* compared with the WT strain. The promoter activity of *waaA* (Figure 3C) was attenuated in Δ*bfvR* relative to the WT, as shown by the β-galactosidase activity assay. EMSA revealed that His-BfvR could bind to the upstream regions of *waaA*- promoter (Figure 3D) in a dose-dependent manner. These results showed that BfvR in *Y. pestis* could promote biofilm formation by directly stimulating the expression of *waaAE-coaD*.

**Figure 4:**
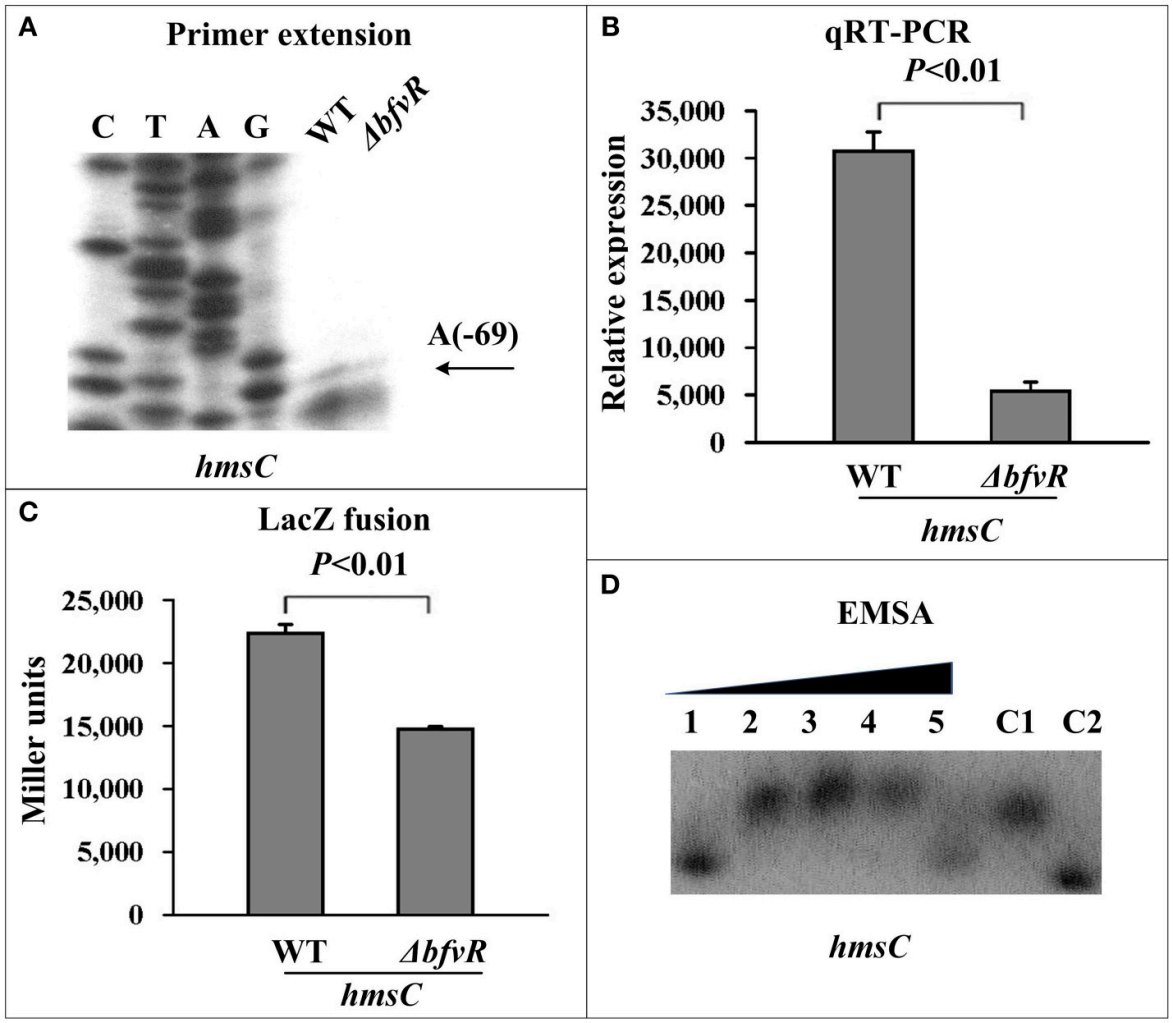
Regulation of *hmsC* by BfvR. Reference to Figure 2 for the annotations of primer extension **(A)**, quantitative RT-PCR **(B)**, LacZ fusion **(C)**, and EMSA **(D)**. The promoter-proximal region from −456 to +55 was amplified.

The c-di-GMP synthesis- and degradation-related genes *hmsT, hmsCDE*, and *hmsP* were also regarded as major targets of biofilm formation at the post-transcriptional level in *Y. pestis*. The mRNA levels and binding activities to His-BfvR of these three genes were investigated by PE, qRT-PCR, β-galactosidase activityassay, and EMSA. Compared withWT, Δ*bfvR* only showed a significant decrease in mRNA levels of *hmsCDE* (Figures 4A–C) but not *hmsT* (Figures S2A–C) or *hmsP* (Figures S3A–C). EMSA also showed that only the promoter-proximal regions of *hmsC* (Figure 4D) but not *hmsT* (Figure S2D) or *hmsP* (Figure S3D) could bind His-BfvR *in vitro*. These results indicated that BfvR in *Y. pestis* could promote c-di-GMP synthesis and regulate biofilm formation by directly stimulating the expression of *hmsCDE*.

### Regulation of the psa loci by BfvR

**Figure 5:**
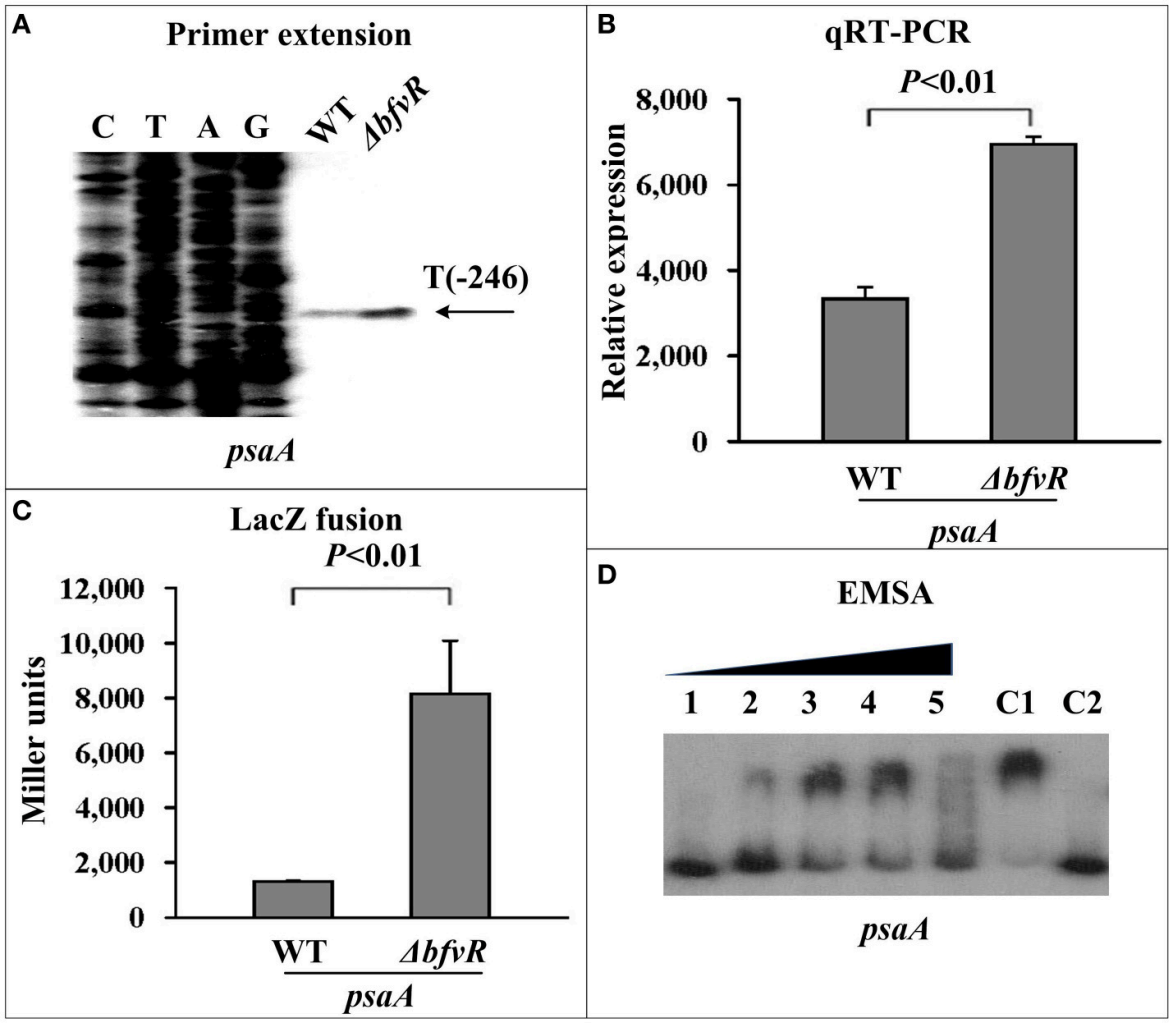
Regulation of *psaA* by BfvR. Reference to Figure 2 for the annotations of primer extension **(A)**, quantitative RT-PCR **(B)**, LacZ fusion **(C)**, and EMSA **(D)**. The promoter-proximal region from −491 to +65 was amplified.

Two loci, *psaABC* and *psaEF* located upstream of *psaA*, responsible for expression of pH 6 antigen (Psa) in *Y. pestis*, were selected as target genes for investigating the regulatory action of BfvR in virulence. PE, qRT-PCR, and β-galactosidase activity assays showed that deletion of *bfvR* led to elevated expression of *psaA* (Figures 5A–C) and *psaE* (Figures S4A–C). EMSA showed that His-BfvR could not bind the promoter-proximal region of *psaE* (Figure S4D) but could bind that of *psaA* (Figure 5D). In our opinion, BfvR might regulate *Y. pestis* virulence through acting on many unknown factors at the same time, but BfvR could at least influence *Y. pestis* virulence by controlling the expression of *psaABC*.

## Discussion

The AraC/XylS family of polypeptides is broadly distributed among Gram negative and positive bacteria (Tobes and Ramos, 2002). Biofilm formation modulation mediated by the AraC-type regulator has been reported for various types of bacteria but, prior to this study, not for *Y. pestis*. In this work, we showed for the first time that BfvR could similarly regulate biofilm formation in *Y. pestis* strain 91001.

Production of extracellular matrix was an important mechanism modulated by the AraC-type transcriptional regulator during biofilm formation. Polysaccharide intercellular adhesin (PIA)/poly-N-acetylglucosamine production was shown to be vital for *Staphylococcus epidermidis* biofilm formation, and this was encoded by the *icaADBC* operon, which in turn was modulated by the AraC-type regulator Rbf (Rowe et al., 2016). In *Enterococcus faecium*, the putative AraC type of regulator EbrB, regulated by *esp* operon expression, was shown to be responsible for the production of the enterococcal surface protein and was implicated in biofilm formation (Top et al., 2013). Another putative AraC-type transcriptional regulator PerA in a clinical isolate of *E. faecalis* strain E99 was detected to be involved in survival within macrophages, pathogenesis in mice, and biofilm formation (Coburn et al., 2008). Similarly, data also showed that BfvR could promote biofilm formation by increasing the synthesis of bacterial EPS and LPS, by directly stimulating the expression of *hmsHFRS* and *waaAE*-*coaD*, respectively.

WaaA plays an important role in the synthesis of lipid A (Dentovskaya et al., 2011) in *Yersinia*, which is responsible for endotoxin and virulence in mice in *Y. pestis* KIM6+ (Sun et al., 2013). It seems to be contradiction that there could be an ~75% reduction in *waaA* gene expression (Figure 3B) and therefore presumably reduced LPS core oligosaccharide production, yet the virulence of the mutant is still enhanced. In fact, the direct effect of WaaA on mice virulence of *Y. pestis* strain 91001 has not been specifically reported. We found that deletion of PhoP or RcsB dramatically induced the downregulation or upregulation of *waaA*, respectively (Liu et al., 2014). However, loss of PhoP or RcsB had no significant effect on *Y. pestis* strain 91001 virulence tested by us (data not shown). In our opinion, downregulation of *waaA* in the BfvR mutant will not necessarily led to a notable alteration of *Y. pestis* strain 91001 virulence in mice. The mechanisms by which WaaA modulates strain 91001 virulence in mice still needs further investigation.

Within current sequence databases, the AraC/XylS family proteins are the most common positive regulators (Tobes and Ramos, 2002). It has been found that deletion of *bfvR* in *Y. pestis* strain 91001 resulted in a dramatic reduction in biofilm production through biofilm-related phenotypic assays. Until now, there has been no report to indicate that c-di-GMP production is regulated by an AraC-family protein. In this study, we demonstrated that deletion of *bfvR* in *Y. pestis* affected the intracellular c-di-GMP levels shown in the biofilm phenotypic assay. Similar to most AraC/XylS family regulators, BfvR displayed positive regulation during biofilm formation in *Y. pestis*.

**Figure 6:**
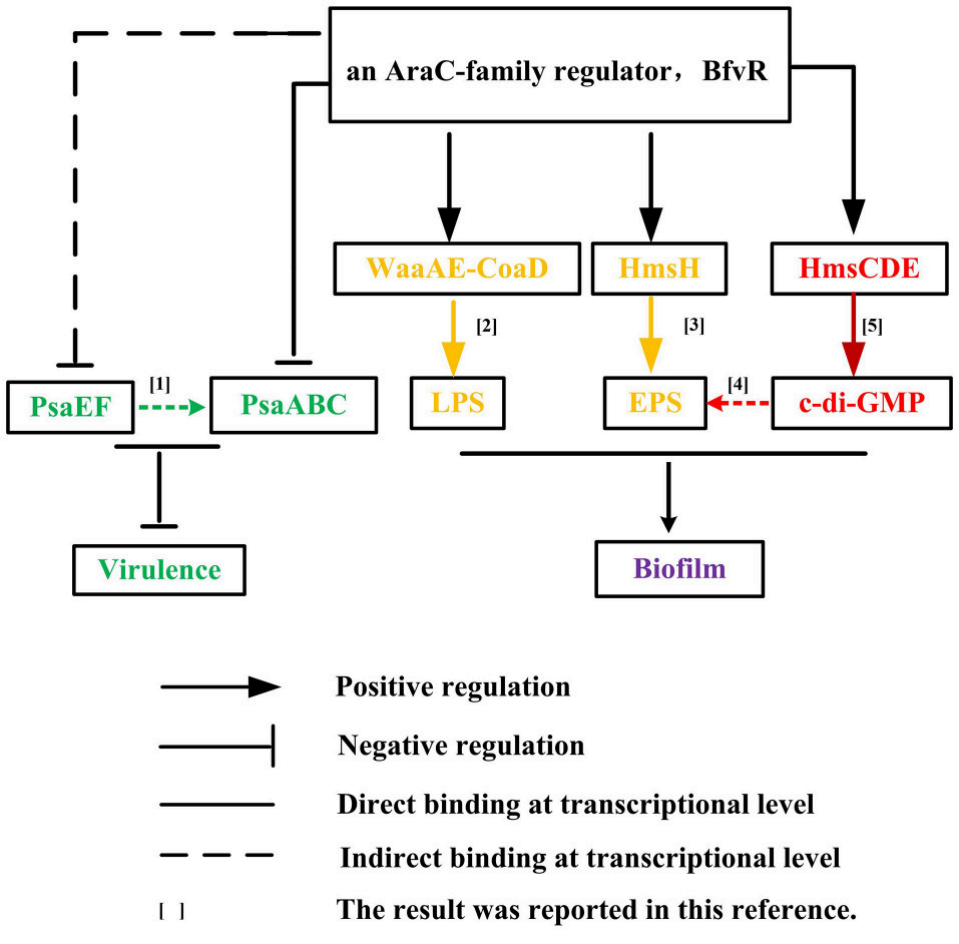
Reciprocal regulation of biofilm formation and virulence by BfvR. The gene regulatory relationship modulated by BfvR was described in the main text and marked in black lines or arrows; while the important clues reported previously were highlighted in other colors. The corresponding literatures were listed as follows:[1] Bao et al. (2012); [2] Tan and Darby (2005); [3] Bobrov et al. (2008); [4] Bobrov et al. (2011); and [5] Kirillina et al. (2004).

The AraC/XylS family of transcription factors have been shown to play an important role in regulating bacterial virulence by modulating the expression of virulence-related genes in *Citrobacter rodentium* (Hart et al., 2008) and *Salmonella enterica* serovar Typhimurium (Bailey et al., 2010). Work reported here also indicated that a similar mechanism existed on virulence modulation by BfvR in *Y. pestis*. BfvR can repress *Y. pestis* virulence in mice by directly and indirectly inhibiting the expression of *psaABC* and *psaEF* at the transcriptional level, respectively. This work further extended our knowledge of regulation mechanisms under the control of the AraC regulator and their biological relevance *in vitro* (Figure 6).

Based on the results of an NCBI conserved domain assay (https://www.ncbi.nlm.nih.gov/Structure/cdd/wrpsb.cgi), the structure of BfvR was found to be homologous to that of SoxS of *Xenorhabdus ishibashii* with 100% coverage and 78% identity aligned by BlastP. SoxS protein was a direct transcriptional activator of the oxidative stress genes of the SoxRS regulon (Li and Demple, 1994), and was widely investigated in strains of family Enterobacteriaceae (Gallegos et al., 1997). SoxS regulated the expression of *ompW* gene, which was involved in osmoregulation as a minor porin, in *Salmonella enterica* serovar Typhimurium (Gil et al., 2009). *ydbK* gene (involved in superoxide resistance) and *ompN* (participating in the minor porins like *ompW*), which were coexpressed in an operon, were indirectly activated by SoxS in a multidrug-resistant *E. coli* strain NorE5 (Fabrega et al., 2012). Redox-cycling drugs rather than superoxide can directly activate the SoxRS response in *E. coli* (Gu and Imlay, 2011). Whether there is a similar SoxRS response shown by BfvR in *Y. pestis* remains to be investigated.

Taken together, our findings suggested that the AraC-family regulator BfvR was firstly found to be involved in the modulation of both biofilm formation and pathogenesis in *Y. pestis* strain 91001. In this study we only focused on biofilm formation and pathogenesis regulated by this regulator, the regulatory role of BfvR in other processes, such as sensing the redox pressure in *Y. pestis*, requires elucidation in future studies.

## Author contributions

DZ and RY conceived the study and designed the experimental procedures. HF, LL, YZ, HY, YY, XD, and YH performed the experiments and carried out data analysis. DZ, HF, LL, YZ, and RY wrote the paper.

### Conflict of interest statement

The authors declare that the research was conducted in the absence of any commercial or financial relationships that could be construed as a potential conflict of interest.
