# Supplementary material for: BfvR, an AraC-Family Regulator, Controls Biofilm Formation and pH6 Antigen Production in Opposite Ways in Yersinia pestis Biovar Microtus
Source: Front Cell Infect Microbiol. 2018 Oct 2;8:347. doi: 10.3389/fcimb.2018.00347 (PMC6176095; doi:10.3389/fcimb.2018.00347)
Supplement: Supplementary file 1 [file Table_1.pdf]

TABLE S1. Oligonucleotide primers used in this study

Primer names	Sequences (5′-3′)
**Construction of mutant**	
1737-K-F	GTGTATACTGCAACACTCCCAACCAGAAAAAACAGACTAAAGATT GCAGCATTACACG
1737-K-R	TAGGCACCTTCTGTATTACCGATGAACAGTGATAACAAGATGTAAC GCACTGAGAAGC
1737-I-F	CTCCCAACCAGAAAAAACAGAC
1737-I-R	ACCGATGAACAGTGATAACAAG
**Construction of complemented mutant**	
1737-C-F	GCGGGATCCGTTGGCAAGCGACCAGAT
1737-C-R	GCGGTCGACCGAAGACATTAAGGAGGA
**Protein expression**	
pColdI-1737-F	TCTGGATCCATGTCACAAGAAAATC
pColdI-1737-R	CATGAAGCTTTCAGGCCTGTTTTTGGT
**Primer extension**	
hmsT-PE-R	GGTATTTATTCCGACATCACGAC
hmsC-PE-R	AGTAGCGGTAGTCATTTTTACG
hmsH-PE-R	TATTGTTGCAAAGTCATTATAGGAT
hmsP-PE-R	CCATCGAGTAAGTTGTGATCC
waaA-PE-R	GTAAACGCAGCCAAATCAGAG
psaA-PE-R	TAACTCAGTCGCAGACCTATAG
psaE-PE-R	TACTGTCACCAATTATTA
YPO1635-PE-R	CAGGGCAAGGGATAATAGGG
phoP-PE-R	ACCCGCATACACCAATCCTT
**LacZ fusion**	
hmsT-LacZ-F/R	GCGGAATTCGCCCAGTACAGGTAACAAGG/GCGGGATCCCTGATCG TAGGAGTGGCTATTC

hmsC-LacZ-F/R	TCTGGATCCCTTACTGGTTGCTATTGCC/TCTAAGCTTGAGGTTCATGATGTTCATCA
hmsH-LacZ-F/R	GCGGGATCCACTTTGCTGAAGACTTGTCACG/GCGAAGCTTCCGCCATAGCAGGATTAACG
hmsP-LacZ-F/R	GCGGGATCCAGCGATGGTAGAAGTGAATCAG/GCGAAGCTTTTGCGATACTCTAATGGAAGGC
waaA-LacZ-F/R	GCGGGATCCTGGATCGCCCAAACATTACG/GCGAAGCTTAAACGCAGCCAAATCAGAGG
psaA-LacZ-F/R	GCGGGATCCGGCGTCTGTCTATATTGGTATC/GCGAAGCTTCCACAAGCAGCGATCATTAG
psaE-LacZ-F/R	GCGGGATCCGTGATCCGATGCGTGTCTTG/GCGAAGCTTTAACAGCAGCACCTCATTCTTG
YPO1635-LacZ-F/R	GCGGGATCCCCGACTCGACCGTGCTAC/GCGAAGCTTATGGCGACACTACAGGAACC
phoP-LacZ-F/R	CCGGAATTCTGATGCCAGCAAAGACG/CGCGGATCCAGATGGTGACGCAACAAC
**qRT-PCR**	
hmsT-RT-F/R	CAGTATGCTATCATCGTCGC/GTAGACCGATGAGGATTG
hmsC-RT-F/R	GGGCGTTTATCTATTCTTAC/GAGTGAGTTATTGGGAAGTG
hmsH-RT-F/R	CTGGCTTTGTCGTTAATCCTG/TGCCCACTCTGCAATGGAC
hmsP-RT-F/R	GGTAAGGCGCTCATTAACGA/TATCAACGCTGAGTATGGCC
waaA-RT-F/R	TTGAACGTGGCGGTCATAAC/GAGGCGGCAATCTTCATCAG
psaA-RT-F/R	GTCAAGCAGGGAAACACATTC/AACCAACATAGTCACCATCGG
psaE-RT-F/R	TGAATTACTGACAACCTGTTGG/TTCGGTGCTGCCATCATC
YPO1635-RT-F/R	GTTCCTGTAGTGTCGCCATG/ACCGCCGTTAAGATAAATCCC
phoP-RT-F/R	ATCTGGAAGAGGTCATTGC/CTGCGTTGCGGATAAGG
**EMSA**	
hmsT-EMSA-F/R	GCCCAGTACAGGTAACAAGG/CTGATCGTAGGAGTGGCTATTC

hmsC-EMSA-F/R	CTTACTGGTTGCTATTGCC/GAGGTTCATGATGTTCATCA
hmsH-EMSA-F/R	ACTTTGCTGAAGACTTGTCACG/ CCGCCATAGCAGGATTAACG
hmsP-EMSA-F/R	GCGGGATCCAGCGATGGTAGAAGTGAATCAG/GCGAAGCTTTTGCGATACTCTAATGGAAGGC
waaA-EMSA-F/R	GCGGGATCCTGGATCGCCCAAACATTACG/GCGAAGCTTAAACGCAGCCAAATCAGAGG
psaA-EMSA-F/R	AAAGCGTCAAATAGCATTGGG/CAGCGATCATTAGTGTGGTAAC
psaE-EMSA-F/R	CCTGTTTGTCCTGCTGATCC/GACTCATTTGCCCTCACCTC
YPO1635-EMSA-F/R	GCGGGATCCCCGACTCGACCGTGCTAC/GCGAAGCTTATGGCGACACTACAGGAACC
phoP-EMSA-F/R	CCGGAATTCTGATGCCAGCAAAGACG/CGCGGATCCAGATGGTGACGCAACAAC
