# Supplementary material for: BfvR, an AraC-Family Regulator, Controls Biofilm Formation and pH6 Antigen Production in Opposite Ways in Yersinia pestis Biovar Microtus
Source: Front Cell Infect Microbiol. 2018 Oct 2;8:347. doi: 10.3389/fcimb.2018.00347 (PMC6176095; doi:10.3389/fcimb.2018.00347)
Supplement: Supplementary file 2 [file Presentation_1.pdf]

BfvR, an AraC-family regulator, controls biofilm formation and pH6 antigen production in opposite ways in *Yersinia pestis* biovar Microtus

Haihong Fang 1, 2†, Lei Liu 3†, Yiquan Zhang 4, Huiying Yang1, Yanfeng Yan1, Xiaojuan Ding5, Yanping Han1, Dongsheng Zhou1#, Ruifu Yang1#

1*State Key Laboratory of Pathogen and Biosecurity, Beijing Institute of Microbiology and Epidemiology, Beijing 100071, China;*

2 *Division of Biology, Beijing Academy, Beijing 100028, China;*

3Department of Blood Transfusion, Wuhan General Hospital of PLA, Wuhan 430070, Hubei, China;

4*School of Medicine, Jiangsu University, Zhenjiang 212013, Jiangsu, China;*

5*Department of Microbiology, Anhui Medical University, Hefei 230032, Anhui, China;*

† Haihong Fang and Lei Liu contributed equally to this work.

Short running title: BfvR controls biofilm and virulence

#**Corresponding authors:**

Ruifu Yang, E-mail: ruifuyang@gmail.com

Dongsheng Zhou, E-mail: dongshengzhou1977@gmail.com

Abstract

Biofilm formation is critical for blocking flea foregut and hence for transmission of *Y. pestis* by flea biting. In this study, we identified the regulatory role of the AraC-family transcriptional regulator BfvR (YPO1737 in strain CO92) in biofilm formation and virulence of *Yersinia pestis* biovar Microtus. Crystal violet staining, *Caenorhabditis elegans* biofilm assay, colony morphology assay, intracellular c-di-GMP concentration determination, and BALB/c mice challenge were employed to reveal that BfvR enhanced *Y. pestis* biofilm formation while repressed its virulence in mice. Further molecular biological assays demonstrated that BfvR directly stimulated the expression of *hmsHFRS*, *waaAE-coaD,* and *hmsCDE*, which, in turn, affected the production of exopolysaccharide, LPS and c-di-GMP, respectively. In addition, BfvR directly and indirectly repressed *psaABC* and *psaEF* transcription, respectively. We concluded that the modulation of biofilm- and virulence-related genes by BfvR led to increased biofilm formation and reduced virulence of *Y. pestis* biovar Microtus.

Keywords: *Yersinia pestis*; BfvR; biofilm; virulence; transcriptional regulation.

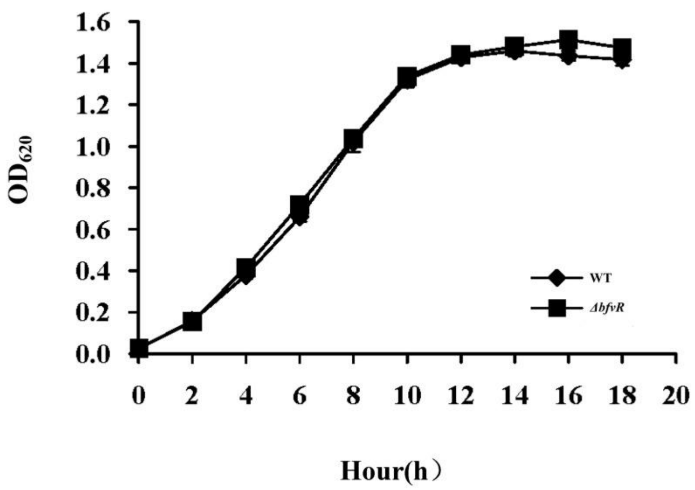

FIGURE S1. Bacterial growth curves. Overnight cell cultures at an OD620 value of approximately 1.0 were diluted 1:20 into 18 ml of LB medium with a pH value of 6.0. Bacteria were then grown at 26 ℃ with shaking at 230 rpm, and the OD620 values were monitored at 3-h intervals until the cell cultures reached the stationary growth phase. Three individual cultures (i.e., three biological replicates) were determined for each strain.

OD 620: Optical density at 620 nm; WT: Wild-type.

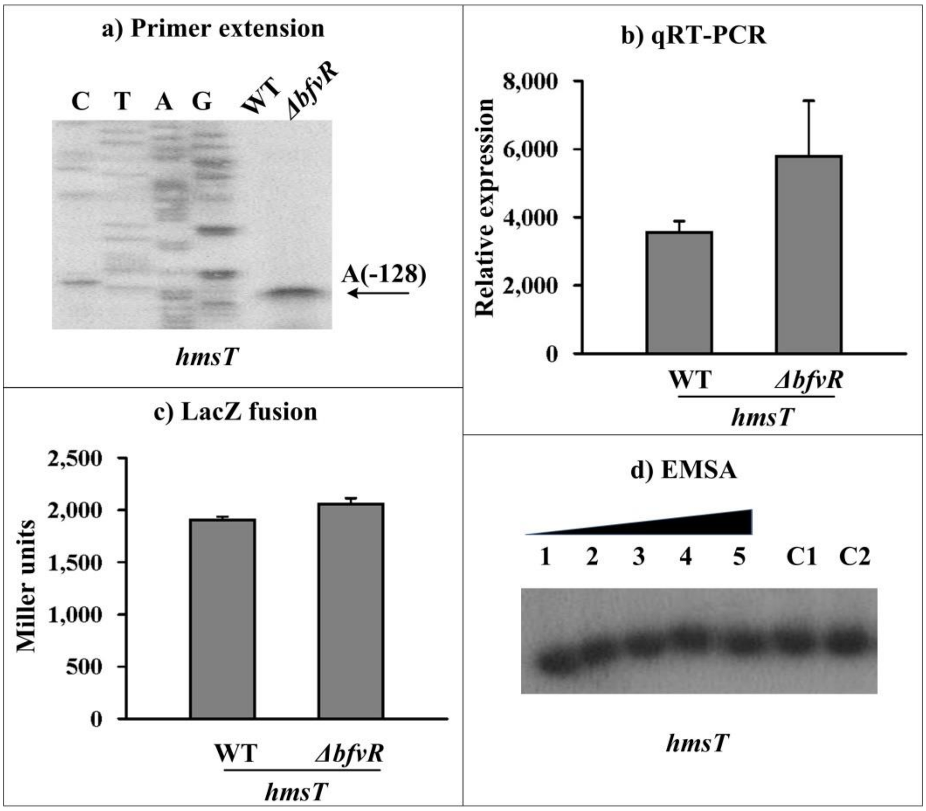

FIGURE S2. Regulation of *hmsT* by BfvR. See FIGURE 2 for the annotations of primer extension (**a**), quantitative RT-PCR (**b**), LacZ fusion (**c**), and EMSA (**d**). The promoter-proximal region was amplified from -410 to +42.

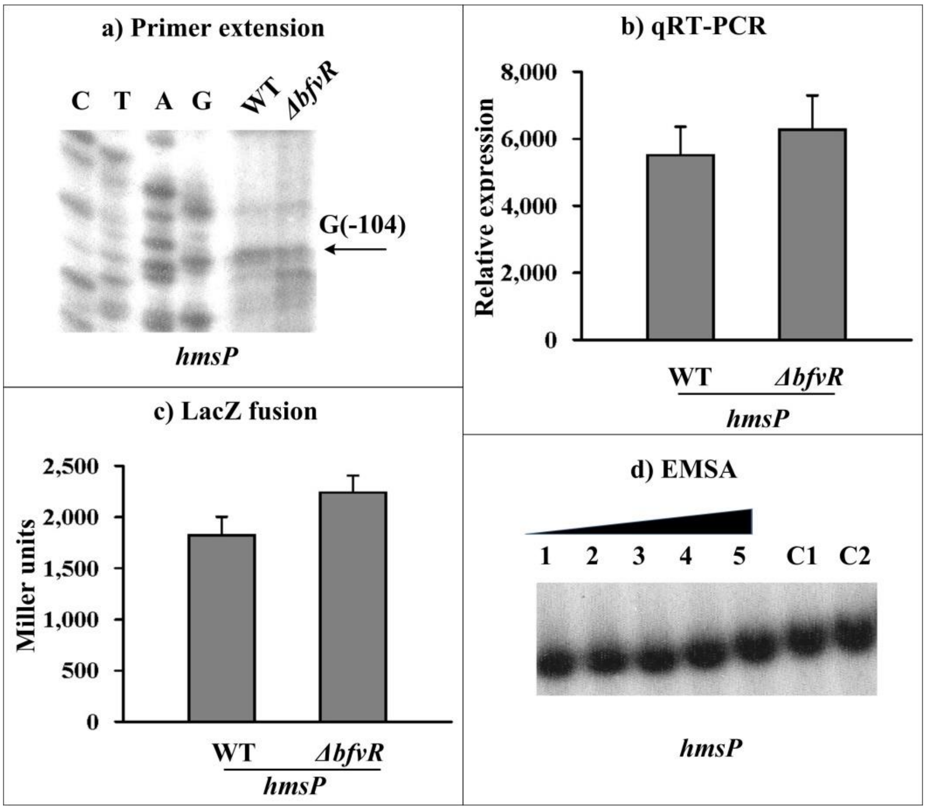

FIGURE S3. Regulation of *hmsP* by BfvR. See FIGURE 2 for the annotations of primer extension (**a**), quantitative RT-PCR (**b**), LacZ fusion (**c**), and EMSA (**d**). The promoter-proximal region was amplified from -659 to +65.

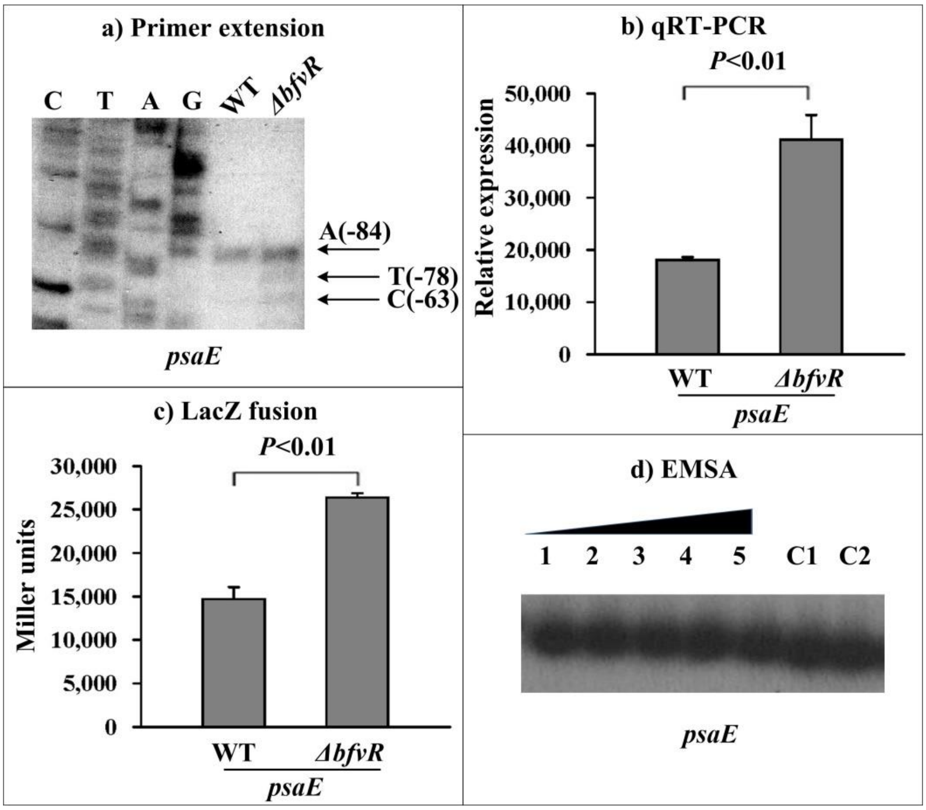

FIGURE S4. Regulation of *psaE* by BfvR. See FIGURE 2 for the annotations of primer extension (**a**), quantitative RT-PCR (**b**), LacZ fusion (**c**), and EMSA (**d**). The promoter-proximal region was amplified from -563 to +93.
