# Supplementary figures and images for: BfvR, an AraC-Family Regulator, Controls Biofilm Formation and pH6 Antigen Production in Opposite Ways in Yersinia pestis Biovar Microtus
Source: Front Cell Infect Microbiol. 2018 Oct 2;8:347. doi: 10.3389/fcimb.2018.00347 (PMC6176095; doi:10.3389/fcimb.2018.00347)

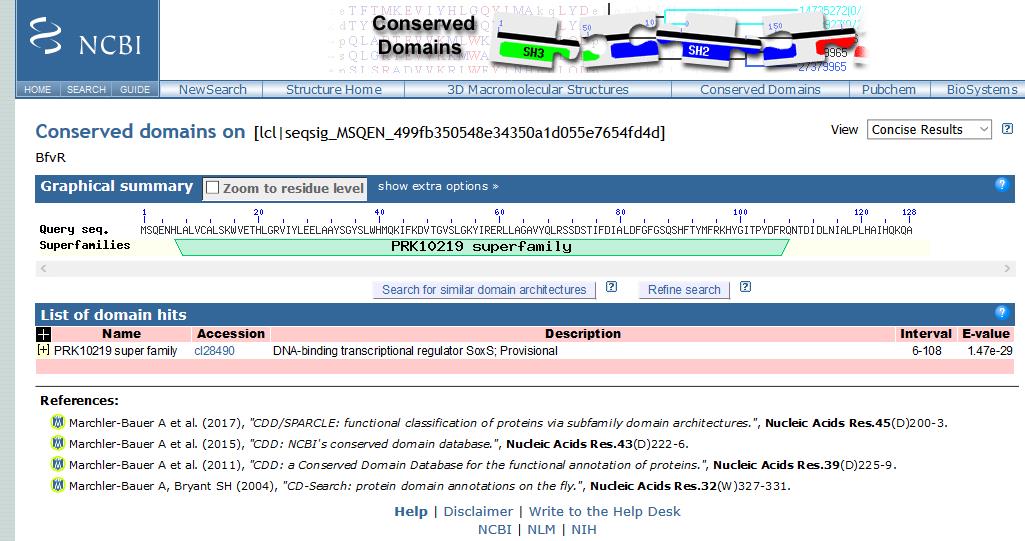

Supplement: Supplementary file 3 [file Image_1.JPEG]

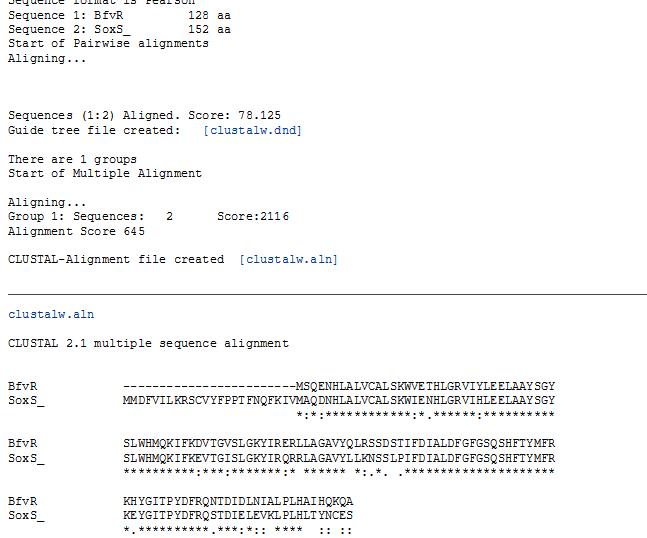

Supplement: Supplementary file 4 [file Image_2.JPEG]
